# Life expectancy of people with hemophilia in France in 2022

**DOI:** 10.1007/s10654-026-01389-z

**Published:** 2026-04-22

**Authors:** Clémence Tabélé, Mohamed Boucekine, Hervé Chambost, Marie-Laure Tiade, Nicolas Giraud, Dorothée Pradines, Yannick Colle, Vincent Dalibard, Djamel Kherfellah, Romain Voltzenlogel, Any Beltran Anzola, Ngoc Anh Thu Nguyen, Noémie Resseguier, Roseline D’Oiron, Dominique Costagliola, Pascal Auquier

**Affiliations:** 1 CEReSS / UR 3279 – Health Services and Quality of Life Research, Aix Marseille University, Marseille, France; 2 FranceCoag Network, Marseille, France; 3 AP-HM, Haemophilia Treatment Centre, Children Hospital La Timone, Marseille, France; 4 Methodological Support Unit for Clinical and Epidemiological Research, University Hospital of Marseille (APHM), Marseille, France; 5 French Patients’ Association for People with Haemophilia (AFH), Paris, France; 6 Lille CHRU, Hemophilia Treatment CenterHematology and Transfusion, Lille, France; 7 Pediatric Oncology Hematology Unit, University Hospital, Bordeaux, France; 8 AP-HP, Haemophilia Treatment Centre, Hospital Bicêtre, Paris, France; 9 Sorbonne University, INSERM, Institut Pierre Louis d’Epidemiology et de Santé Publique, Paris, France

**Keywords:** Hemophilia, HIV, Innovative therapy, Insurance, Life expectancy

## Abstract

The life expectancy of adult people with hemophilia has significantly increased thanks to therapeutic advancements, especially after the bloodborne infectious risks were controlled. However, no French data has yet documented this improvement. This study aims to compare the life expectancy of French people with hemophilia to that of the general population. A longitudinal study was conducted on 9,928 people with hemophilia included in the FranceCoag database between 1994 and 2022. Life expectancies were calculated using abridged life tables The life expectancies for people with hemophilia were 66.3 years at age 20 (mild hemophilia = 68.8, moderate = 64.8, severe = 60.4) and 47.5 years at age 40 (mild = 49.2, moderate = 45.9, severe = 42.7). Individuals with mild or moderate hemophilia had higher life expectancies compared to general population (59.8 years at age 20 and 40.7 years at age 40, *p* < 0.001). Those with severe hemophilia had a similar or slightly higher life expectancy at age 40. The mean differences in LE at ages 20 and 40, compared with the general population, varied markedly by severity, ranging from 0 to 10 years. Taken together, these findings suggest broadly comparable life expectancy in people with hemophilia, although differences remain according to disease severity. This improvement may be attributable both to recent therapeutic innovations and to more frequent and comprehensive medical follow-ups compared to the general population.

**Supplementary Information:**

The online version contains supplementary material available at 10.1007/s10654-026-01389-z.

## Introduction

Constitutional hemophilia is a rare X-linked inherited disorder that results in a coagulation activity defect (factor VIII (FVIII) in hemophilia A or factor IX (FIX) in hemophilia B). The result is spontaneous or post-traumatic hemorrhagic manifestations, more or less frequent depending on the severity of the deficiency [1]. In 2019, worldwide prevalence was estimated at 17.1 (14.8–19.3) in hemophilia A and 3.8 (3.2–4.4) in hemophilia B per 100000 men [2]. The annual 2022 report of FranceCoag, a national monitored registry and research organization dedicated to people living with a constitutional bleeding disorder in France, includes 7892 people with hemophilia (PWH A and 1760 with hemophilia B [3].

Until the 1980 s, hemophilia treatment relied on human plasma derived clotting factor concentrates, which were responsible for the transmission of pathogens like Human Immunodeficiency Virus (HIV), Hepatitis C Virus (HCV), Hepatitis B Virus (HBV) and had a major impact on the mortality of people with hemophilia (PWH). Recombinant factors have since taken over [4], limiting the risk of infection, and more recently, the availability of new innovative therapies such as extended half-life recombinant factors or non-replacement therapies, (monoclonal antibodies or first administrations of gene therapies) have improved the quality of life of PWH [4]. Developments of treatment of infectious diseases have also improved the vital prognosis of PWH living with HIV or HCV [5–9]. This led to favorable trends in life expectancy (LE) for PWH in various countries with well-established hemophilia care programs and access to treatment: in the UK, LE at birth was between 63 and 75 years in 1999, depending on the severity of hemophilia [10]; in the Netherlands, it was 73 to 80 years in 2018 [11]. However, there is currently no published data on the LE of PWH in France.

In recent decades, one of the challenges faced by PWH in France is the difficulty in accessing property ownership, the underwriting of which is closely linked to obtaining loan insurance, which they are often unable to benefit from [12]. Since 2015, the French government has signed a contract with insurers setting out the conditions under which people with specific chronic diseases could take out an insurance policy with or without a surcharge, or exclusion of cover as part of a bank loan. After examination, the chronic diseases are registered on the AERAS reference grid (details in supplementary data) as appendix of this contract, with an annual update of eligible pathologies – provided that therapeutic progress and scientific data attest to the capacity of the treatments concerned to significantly and durably limit their effects. HIV was included in the AERAS grid based on the improved life expectancy of people living with the infection, which is now close to that of the general population – despite initial concerns from insurers about its potential impact on life expectancy and ability to work [12–14]. As was done for people living with HIV, the inclusion of PWH on the AERAS grid represents a major societal issue, aiming to reduce inequalities for this population affected by a rare bleeding disorder.

In light of the findings outlined above, it is essential to have an up-to-date and accurate understanding of the LE of PWH in France. This information is crucial not only for evaluating the long-term impact of the numerous medical and therapeutic advances, but also for informing public policy, improving patient support, and addressing issues such as access to insurance and credit. Indeed, it would be a key factor in including PWH in the AERAS grid, thereby supporting changes to the rules governing their access to loans in the country.

The primary objective of this study is to assess the LE of PWH in France in 2022, both at birth and at adult age, and to compare it with that of the general French population. Secondary objectives include comparing LE at typical borrowing age with that of people living with HIV, and with the typical duration of a bank loan, in view of potential inclusion on the AERAS grid.

## Methods

### Design

To meet the objectives, an observational, prospective, longitudinal, descriptive, multicenter study was set up.

### Data sources

Data from the FranceCoag system (epidemiological tool of the Filière Maladies Rares MHEMO, built up from the registration of personal data of people living in France and affected by constitutional hemorrhagic diseases by 34 reference centers throughout France identified by MHEMO) were used to evaluate LE of PWH [3].

LE estimated in this study were then compared with data from other sources. PWH LE were compared with LE of the general French population: data from the Institut National de la Statistique et des Études Économiques (INSEE) (based on the entire French population) [15]. PWH LE were compared with LE of people living with HIV: data from the Trickey study (work resulting from the collaboration of several European and American cohorts) [13]. PWH LE were compared with the duration of bank loans in France: data from the Journal Officiel de la République Française (JORF) [16].

### Population

**Fig. 1:**
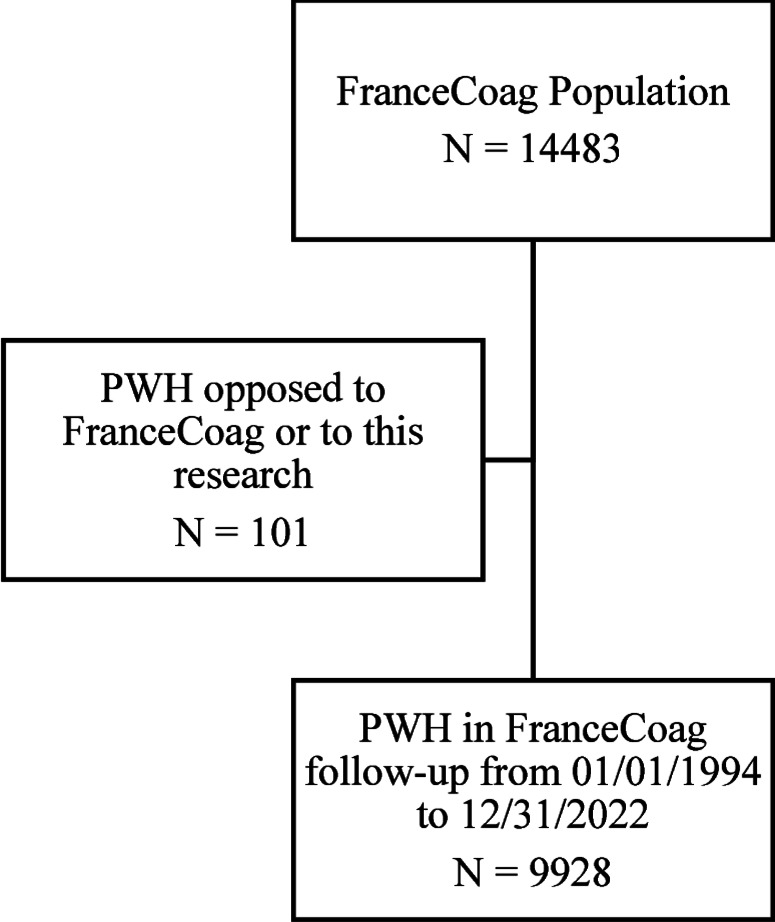
Flowchart for inclusion of people with hemophilia (PWH)

The source population was drawn from the FranceCoag system, followed up between January 1, 1994 and December 31, 2022, which represents approximately 80% of French PWH overall. Coverage is close to exhaustive for moderate and severe PWH, who are routinely managed in specialized hemophilia treatment centers, whereas mild PWH are less systematically followed and therefore underrepresented. The entire eligible FranceCoag active file was enrolled in the study (Fig. 1), including PWH living with HIV and/or HCV.

### Data collected (details in supplementary data)

#### Data collected from participants

In order to meet the study objectives, the following data were collected from participating individuals: sex, age at last visit, vital status, severity of hemophilia, diagnosis circumstances, recorded infections at inclusion or during follow-up (HIV, HCV, HBV), liver diseases excluding liver infection at inclusion or during follow-up, prophylaxis and onset of inhibitors at inclusion or during follow-up, severe bleeding during follow-up (intracranial and other life-threatening bleedings), initiation of factor replacement treatment during the period at risk of transmission of blood-borne viruses (until 1990), and total numbers and deaths.

#### Reference data

Other data came from reference publications : LE for the general French population in 2022 and for people living with HIV as presented in the Trickey et al. 2023 study [13] (with CD4 count > 500 cells/µL, initiation of antiretroviral treatment after 2015 and undetectable viral load).

### Statistics (details in supplementary data)

Statistical analyses were mainly carried out using R software (version 4.3.3).

Descriptive statistics were carried out according to the type of variable. Continuous variables were summarized in median and interquartile ranges or means and confidence intervals 95%CI (for LE). Categorical variables were described in terms of numbers and frequencies. Corresponding confidence intervals and p-values were obtained using Bootstrap resampling techniques [17, 18].

An underestimation of severe and moderate hemophilia cases is possible as some may not be followed-up in centers participating in FranceCoag. This is even more plausible for cases of mild hemophilia. To limit this underestimation and to limit the biases associated with the inclusion of both prevalent and incident cases, a data adjustment technique based on type of severity and age category was applied, using weighting coefficients taken from the Canadian Hemophilia Registry (CHR) [19]. The CHR registry was chosen for 2 reasons: 1/the registry has been shown to be exhaustive in counting the population with hemophilia in Canada; 2/the level of access to care in Canada is similar to that in France. There is therefore no argument in favor of a different distribution of PWH in Canada and France [20–22]. Weighting coefficients were calculated and used to adjust the numbers of PWH in the FranceCoag registry by age group and severity.

Similarly, there may be an underestimation of deaths among people lost to follow-up, due to unreported deaths. In the FranceCoag system, after searching for the deceased among a sample people lost to follow-up, this rate is 19%, which is consistent with the literature, generally reporting death rates ranging from 9% to 36% among people lost to follow-up, depending on the type of chronic disease [23–27]. Then a multivariate logistic regression model was used to predict death based on demographic and clinical characteristics (sex, age, severity of hemophilia, infections, non-infection liver disease, onset of inhibitor, severe bleeding, initiation of treatment during the contaminated blood period, before 1991, time between last FranceCoag visit and today or death, circumstances of diagnosis): risk-of-death coefficients were applied for each PWH lost to follow-up. Finally, among PWH lost to follow-up in the entire FranceCoag database, the 19% (death rate found in a sample of people lost to follow-up in FranceCoag) who had the highest risk-of-death coefficients were considered to have died. To evaluate the validity of the imputation model for PWH lost to follow-up, we assessed model discrimination (area under the receiver operating characteristic (ROC) curve) and calibration (calibration intercept and slope), as well as overall predictive accuracy using the Brier score and pseudo-R² indices. In addition, sensitivity analyses were performed by applying alternative mortality assumptions among lost-to-follow-up PWH (including a higher death proportion of 30%) to examine the impact on LE estimates.

As for the Trickey et al. 2023 study [13], abridged mortality tables were then constructed using age-specific mortality rates per 1000 person-years, as the total number of deaths divided by the total number of person-years of observation (time between date of birth and date of last news, such as date of death or date of last visit to the hemophilia center) during the study period. Abridged rather than complete annual life tables were used to ensure stability of age-specific mortality rates given the sample size. It allowed to estimate expected remaining years of life (LE), as period life expectancy, stratified by severity (mild, moderate and severe hemophilia) and age group for PWH in 2022. The abridged mortality tables constructed for these analyses were based on standardized methods used in previous studies [28, 29].

LE comparisons with people who have contracted HIV were made on the basis of data published by Trickey et al. in [13], the AERAS working group having itself used this study to include HIV on this grid, in addition to criteria other than LE. Life expectancies comparisons were made at birth, and at ages 20 and 40 – these latter ages were selected in accordance with Trickey study [13]. However, LE data for all available age groups are provided in the supplementary data. Finally, comparisons were made with the general male population, since hemophilia primarily affects men.

An additional analysis was also carried out excluding people who contracted HIV or HCV during the contaminated blood period (the 2010 threshold was chosen to take into account delays in diagnosis and/or declaration in FranceCoag), to understand whether this factor might be linked to a different LE in this FranceCoag sub-population.

The methods for estimating LE are described in more detail in the supplementary data.

### Ethics

FranceCoag received a favorable opinion from the CNIL in December 2020 (Avis N° 2218579) as a certified health data hosting provider. Participating subjects were informed of their participation individually at the time of their inclusion in the FranceCoag system, and by general means (https://www.francecoag.org/SiteWebPublic/html/accueil.html) for this study of the nature of the information processed, its purpose and the identity of the natural and legal persons receiving the data. The study was based on the principle of information and non-opposition: in the absence of any opposition, people eligible for the study were included.

## Results

### Population description

A total of 9928 PWH were included in the study. Table 1 presents their demographic and clinical characteristics. PWH constituted a population with a median age of 33.0 years, which is lower than the median age in the general population (over 40 years) [15]. The sex ratio showed male predominance, in line with the mode of transmission of the disease (sex ratio = 13.7). More than one person in 5 have been affected with an infectious disease such as HIV, HCV or HBV.

**Table 1:** Demographic and clinical characteristics of people with hemophilia

Variables	PWH *N* = 9928 *n* (%)	Severe* N = 2843 *n* (%)	Moderate* N = 1460 *n* (%)		Mild* N = 5621 *n* (%)	*p*-value
**Age at last visit (in years)**						**< 0.001**
Median (IQR)	33.0 (17–52)	32 (17–48)	34 (18–55)		33 (17–54)	
**Sex**						**< 0.001**
Male	9252 (93.0)	2,832 (100)	1,446 (99.0)		4,970 (88.0)	
Female	676 (6.8)	11 (0.4)	14 (1.0)		651 (12.0)	
**Age at diagnosis**						**< 0.001**
Median (IQR)	3 (1–16)	1 (0–1)	2 (0–7)	10 (3–28)		
**Diagnosis circumstances**						**< 0.001**
Screening due to family history	3373 (34.5)	781 (28.5)	601 (42.0)	1988 (35.5)		
Secondary to a bleeding event	4001 (40.9)	1688 (61.5)	652 (45.6)	1660 (29.7)		
Fortuitously during a routine hemostasis test	1937 (19.8)	81 (2.9)	110 (7.7)	1746 (31.2)		
Unknown	465 (4.8)	195 (7.1)	68 (4.7)	202 (3.6)		
Missing data	152	98	29	25		
**Infection at inclusion or during follow-up (**HIV, HCV, HBV)						**< 0.001**
Yes	2280 (23.0)	1,173 (41.0)	478 (33.0)		628 (11.0)	
No	7648 (77.0)	1670 (59.0)	982 (67.0)		4993 (89.0)	
**Liver disease other than infection at inclusion or during follow-up**						**< 0.001**
Yes	84 (0.8)	38 (1.3)	18 (1.2)		28 (0.5)	
No	9844 (99.2)	2805 (98.7)	1442 (98.8)		5593 (99.5)	
**Severe bleeding during follow-up**						**< 0.001**
Yes	663 (6.7)	380 (13.0)	131 (9.0)		151 (2.7)	
No	9265 (93.3)	2463 (87.0)	1329 (91.0)		5470 (97.3)	
**Occurrence of inhibitors at inclusion or during follow-up**						**< 0.001**
Yes	809 (8.1)	624 (21.9)	78 (5.3)		107 (1.9)	
No	9119 (91.9)	2219 (78.1)	1382 (94.7)		5514 (98.1)	
**Prophylaxis during follow-up**						**< 0.001**
Yes	2995 (30.0)	2506 (88.0)	366 (25.0)		121 (2.2)	
No	6933 (70.0)	337 (12.0)	1094 (75.0)		5500 (97.8)	
**Initiation of replacement therapy during period at risk of transmission of blood-borne viruses **(until 1990)						**< 0.001**
Yes	1574 (16.0)	822 (29.0)	333 (23.0)		418 (7.4)	
No	8354 (84.0)	2021 (71.0)	1127 (77.0)		5203 (92.6)	

*four participants with unknown severity of hemophilia

*PWH* People with hemophilia, *HIV* Human immunodeficiency virus, *HCV* hepatitis C virus, *HBV* hepatitis B virus

Terms in bold indicate a statistically significant difference

Severe hemorrhage and infections were predominant in severe form of hemophilia, as was the use of prophylactic regimens or the development of inhibitors against FVIII or FIX. There were thus several statistically significant differences between people with severe, moderate and mild hemophilia, which meant that LE analyses stratified by severity had to be carried out.

### LE estimates for PWH

LE at age 20 and 40 for PWH, calculated from abridged life tables, are shown in Table 2.

**Table 2:** People with hemophilia life expectancies at birth, ages 20 and 40

	At birth	At age 20	At age 40
**All PWH**, Mean (95%CI)	69.2(59.1–76.8)	66.3(66.0–66.6.0.6)	47.5(47.2–47.7)
**PWH and without HIV/HCV before 2010**, Mean (95%CI)	68.5 (59.5–77.0)	67.2 (66.8–67.5)	47.8 (47.4–48.1)

*PWH* People with hemophilia; HCV: hepatitis C virus; HBV: hepatitis B virus

LE at age 20 for PWH increased by around one year when people who contracted HIV or HCV during the period of high patient exposure to contaminated blood (before 2010) were excluded from the hemophilia population.

LE estimates for hemophilia patients at ages 20 and 40, stratified by severity are reported in Table 3.

**Table 3:** People with hemophilia life expectancies at birth, and at 20 and 40, according to severity of hemophilia

	Severe *N* = 2843	Moderate *N* = 1460	Mild *N* = 5621
**LE (95% CI) at birth**			
LE (in years), Mean (95%CI)	46.3 (27.5–64.1)	71.8 (33.1–85.5)	85.4 (81.1–88.5)
LE mean difference (in years) : [LE(i)-LE(n)]*	−39.1	−13.5	–
p-value LE difference**	< 0.001	0.292	–
**LE (95% CI) at age 20**			
LE (in years), Mean (95%CI)	60.4 (59.6–61.3)	64.8 (63.9–65.7)	68.8 (68.5–69.2)
LE mean difference (in years) : [LE(i)-LE(n)]*	−8.4	−4.0	–
p-value LE difference**	< 0.001	< 0.001	–
**LE (95% CI) at age 40**			
LE (in years), Mean (95%CI)	42.7 (41.9–43.4)	45.9 (45.2–46.6)	49.2 (49.0–49.5.0.5)
LE mean difference (in years) : [LE(i)-LE(n)]*	−6.5	−3.3	–
p-value LE mean difference**	< 0.001	< 0.001	–

*LE (i) = LE for indicated hemophilia severity; LE(n) = LE for mild hemophilia (reference)

i.e.: at 20 years, LE for moderate PWH is on average 4 years shorter than for mild PWH

**p-value LE difference: statistically significant difference vs. mild hemophilia

*PWH* People with hemophilia, *LE* Life Expectancies

At ages 20 and 40, the LE of PWH increased with decreasing severity: thus, at age 20, a mild PWH can expect to live 8 years longer than a severe PWH. Mean LE at birth were 46.3 (95% CI: 27.5–64.1) years for severe hemophilia, 71.8 (95% CI: 33.1–85.5) for moderate hemophilia and 85.4 (95% CI: 81.1–88.5) for mild hemophilia.

### Comparison of LE for PWH and for the general population or the HIV population in France

**Fig. 2:**
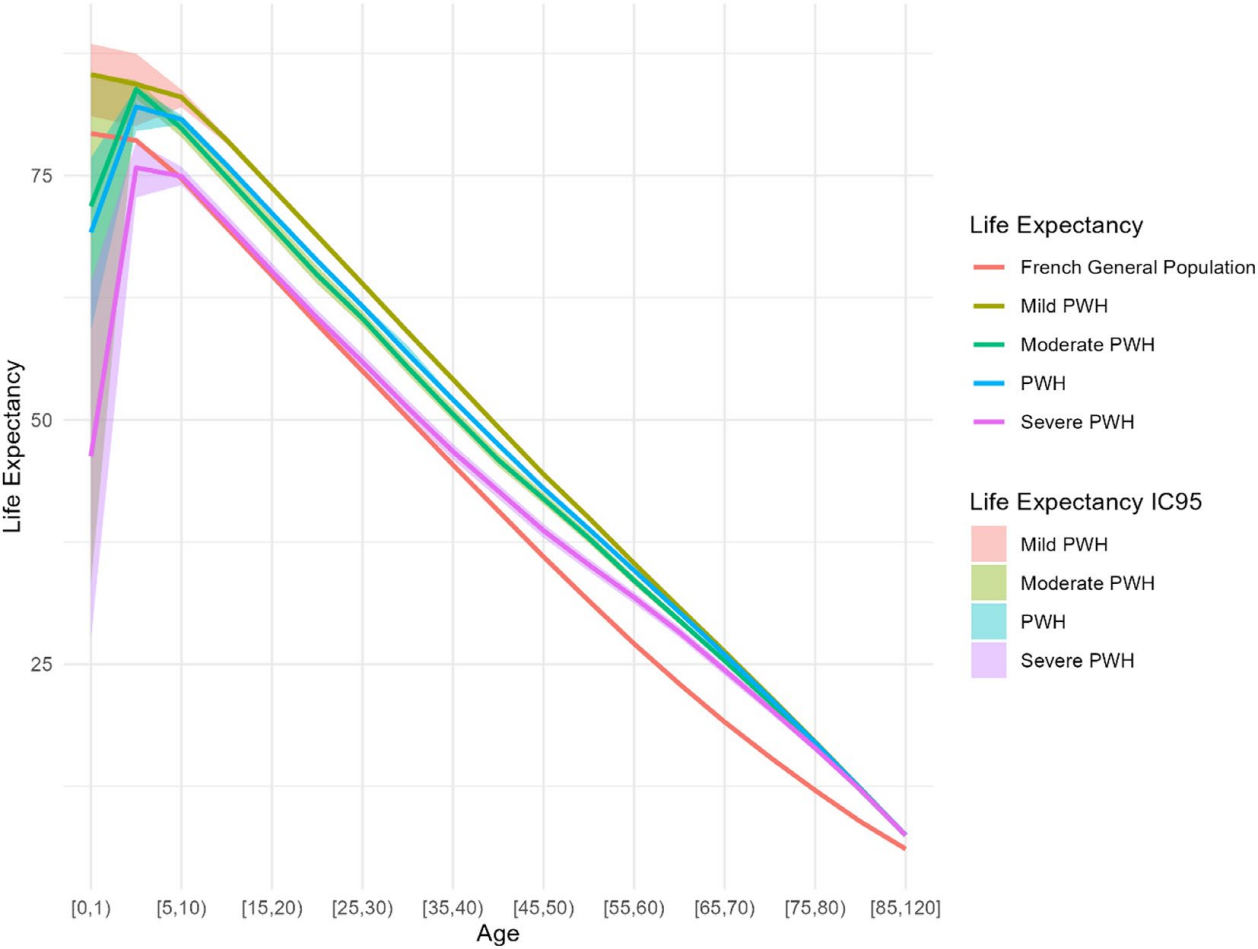
Hemophilia life expectancy (LE) stratified by severity

At ages 20 and 40, LE in adults PWH consistently exceeded 40 years across all severity levels. For example, even the lowest observed mean LE substantially exceeded a 25-year benchmark, highlighting that survival prospects in this population are now markedly extended (*p* < 0.001). LE at age 20 and 40 for PWH compared with the French population or people living with HIV, are reported in Table 4. Overall, the LE of total haemophilia adult patients at 20 and 40 years, were statistically higher than those of the general population. After stratification according to severity, they remained higher for mild and moderate hemophilia patients (Fig. 2). In patients with severe hemophilia, LE was statistically higher at age 40 than in the general population, but at age 20 the difference was not statistically significant. However, life expectancies for severe PWH were all significantly higher than those for the general French population from the age of 25, with an increasingly strong link with the passing years (details in supplementary data). In terms of absolute magnitude, mean differences in LE compared with the general population varied markedly by severity: they were small for severe hemophilia (0–1.5 years at age 20 and 1–2 years at age 40), intermediate for moderate hemophilia (4–6 years at both ages), and substantial for mild hemophilia (approximately 9–10 years at age 20 and around 9 years at age 40). Overall, the LE of haemophilia patients were statistically higher than those of patients living with HIV at 20 and 40 years. After stratification by severity, these differences remained statistically higher.

**Table 4:** Comparisons of people with hemophilia life expectancies at birth and at ages 20 and 40

	Total	Severe	Moderate	Mild
**LE (95% CI) at birth**				
PWH (in years), Mean (95%CI)	69.2 (59.1–76.8)	46.3 (27.5–64.1)	71.8 (33.1–85.5)	85.4 (81.1–88.5)
General population (in years)	79.3			
Mean				
p-value	**0.016**	**0.002**	0.652	**0.018**
**LE (95% CI) at age 20**				
PWH (in years), Mean (95%CI)	66.3 (66.0–66.6.0.6)	60.4 (59.6–61.3)	64.8 (63.9–65.7)	68.8 (68.5–69.2)
People with HIV (in years), Mean (95%CI)	58.5 (58.0–59.0)			
p-value	**< 0.001**	**< 0.001**	**< 0.001**	**< 0.001**
General population (in years)	59.8			
Mean				
p-value	**< 0.001**	0.144	**< 0.001**	**< 0.001**
**LE (95% CI) at age 40**				
PWH (in years), Mean (95%CI)	47.5 (47.2–47.7)	42.7 (41.9–43.4)	45.9 (45.2–46.6)	49.2 (49.0–49.5)
People with HIV (in years), Mean (95%CI)	39.2 (38.7–39.7)			
p-value	**< 0.001**	**< 0.001**	**< 0.001**	**< 0.001**
General population (in years)	40.7			
Mean				
p-value	**< 0.001**	**< 0.001**	**< 0.001**	**< 0.001**

*PWH* People with hemophilia, *HIV* Human immunodeficiency virus

Terms in bold indicate a statistically significant difference

### Performance of the imputation model (details in supplementary data)

The imputation model for PWH lost to follow-up demonstrated strong predictive performance. Discrimination was excellent, with an AUC (C-statistic) of 0.895 (95% CI: 0.883–0.907), indicating a high ability to distinguish between deceased and non-deceased PWH. Calibration was satisfactory, with a calibration intercept close to zero (− 0.066) and a slope near one (0.967), suggesting minimal systematic bias and limited overfitting. Calibration errors were small (Emax = 0.036; Eavg = 0.005), and the Brier score (0.062) confirmed good overall accuracy. Model fit indices further supported its robustness: McFadden’s R² was 0.382, while Cox & Snell and Nagelkerke R² values were 0.23 and 0.464, respectively, indicating substantial explanatory power. Tjur’s R² (0.314) reflected good separation between predicted probabilities in deceased and surviving PWH. Overall, these metrics indicate strong discrimination, adequate calibration, and robust explanatory performance within the study cohort.

### Sensitivity analysis (details in supplementary data)

Sensitivity analyses using a higher assumed mortality rate among PWH lost to follow-up (30% vs. 19% in the primary analysis) showed minimal impact on LE estimates. Differences were ≤ 1 year in mild PWH, null in moderate PWH, and limited to − 2 to + 2 years across ages in severe PWH. These modest variations support the overall robustness of our LE estimates to plausible imputation assumptions.

## Discussion

To the best of our knowledge, this is the first and most comprehensive study in France to provide life expectancy data for adults with hemophilia. It showed a favorable trend as they had a high overall LE, which also varied significantly according to the severity of the hemophilia, in line with therapeutic advances in the treatment of hemophilia.

In 1980, before the period of risk related to the transmission of blood-borne viruses, Larson et al. [30] estimated the median LE (i.e. LE at birth) among PWH to range from 56.8 to 72.1 years, depending on disease severity. Approximately twenty years later, Darby et al. in England reported a median LE of 63–75 years, again depending on severity, although their analysis excluded PWH who were HIV-positive [10, 31]. More recently, Hassan et al. (2018) in the Netherlands reported a median LE of 79–80 years, according to severity [11]. The progressive improvement in LE over time may be attributed to enhanced safety of plasma-derived products, the introduction of recombinant clotting factors produced using genetically engineered cells, and advances in recombinant technology [1]. These innovations have substantially reduced the risk of viral transmission in PWH. n addition, improved bleeding control, wider use of prophylaxis in severe and moderate hemophilia, advances in antiretroviral therapy and HCV eradication treatments, and improved management of inhibitors (including immune tolerance induction and more recently emicizumab) have likely contributed to increased LE.

Comparisons with historical studies should be interpreted cautiously due to important methodological differences. Earlier studies primarily reported median LE at birth, whereas the present study, as well as that by Trickey et al. [13] in 2023, estimated mean LE in adults (at ages 20 and 40). Estimates at birth are not directly comparable to adult life expectancy, as they are typically derived from survival analyses, whereas our study used abridged life tables adapted to adult age groups and accounting for age-specific mortality patterns.

Additional differences arise from variations in study periods, population characteristics (with earlier studies often including only males), exclusion of HIV-positive individuals [10], and smaller sample sizes (approximately 1,000 PWH in Swedish and Dutch registries).

At last, lower LE estimates in our study at birth may also be due to death peak in children under one year of age in severe and moderate PWH, i.e. 500.0 and 166.7 deaths per 1000 Person-year respectively. This excess mortality is thought to be linked to the intracranial hemorrhages to which children with haemophilia, especially severe ones, are exposed at birth an early age prior initiation of prophylaxis, and to the associated high neonatal mortality [10, 32]. Thus, in severe and moderate PWH, the burden of the disease at the earliest ages may have an impact on LE. However, the absence of recorded deaths among mild PWH under one year of age, resulted in a zero infant mortality rate. This likely reflects delayed diagnosis (median 7.4–7.8 years), whereby mild PWH who died before the clinical diagnosis of hemophilia were not all captured in the registry, potentially leading to an overestimation of LE compared with the French general population.

In our study, LE at ages 20 and 40 among adults with severe hemophilia differed from that of the general French population by only 0–2 years. Although some comparisons reached statistical significance, these differences fall below the 2–5-year range generally considered clinically meaningful in public health contexts. This suggests that, beyond early excess mortality, adults with severe hemophilia now achieve a life expectancy that is effectively comparable to that of the general population — a finding that may reflect the major progress made in hemophilia management over recent decades. For moderate hemophilia, LE differences ranged from approximately 4 to 6 years, and for mild hemophilia from 9 to 10 years. While these magnitudes reach or exceed commonly proposed thresholds for clinical relevance, they must be interpreted in context: the observed survival levels represent a marked improvement compared with historical data and indicate that most PWH now experience long-term survival well into older adulthood. Rather than signaling a widening gap, these findings highlight the considerable gains achieved across all severity groups. Importantly, these comparisons are made against the general French population, which includes individuals living with chronic diseases. According to INSEE, approximately one in three people in France lives with a chronic condition [33]. Life expectancy at birth in a chronic disease–free French population is estimated between 91 and 94.8 years [33], substantially higher than that observed in the overall population. When compared with such a disease-free reference group, life expectancy in people with mild, moderate, or severe hemophilia remains lower across all severities, highlighting that, despite major therapeutic advances, hemophilia continues to be associated with a measurable long-term survival gap. On the other hand, PWH benefit from a better follow-up, with earlier medical and surgical care pathway of their pathology and all associated co-morbidities. Their more frequent and thorough medical follow-up than in the general population could also be one of the reasons for this difference in LE [34]. Evidence regarding cardiovascular disease (CVD) risk in PWH remains inconsistent. While some studies report a lower prevalence of arterial thrombotic events compared with the general population, others show comparable or even higher rates [35–37]. Although reduced coagulability has been hypothesized to confer partial protection against fatal ischemic heart disease, current data are insufficient to draw firm conclusions. Further prospective and longitudinal studies are needed to better characterize CVD risk and optimize prevention strategies in this population.

Our study showed that LE in PWH has improved to levels similar to the general population, suggesting potential relevance for long-term social and financial planning [16]. This supports the consideration of hemophilia in the AERAS grid [12], reflecting the substantial gains in survival achieved in this population.

The LE of hemophilia patients is higher than that of patients living with HIV at 20 years and 40 years, regardless of severity. This can be explained by two phenomena: firstly, PWH were no longer overexposed to HIV and other infections such as HCV and HBV. Secondly, exposed patients benefited from therapeutic innovations in the treatment of both these viral infections [9] and hemorrhagic diseases [1]. As a result, the double burden of mortality that contributed to reducing their LE was largely reduced. What’s more, despite their severe hemorrhagic phenotype [38], people with severe hemophilia had higher LE at 20 and 40 years than those living with HIV (and with a CD4 count above 500/µl) [13]. This means that PWH, whatever their severity, could qualify for inclusion in the AERAS grid, given the higher LE reported in comparison with HIV, a disease already included in the grid.

Our study presents few limitations. FranceCoag does not fully capture all mild PWH, as some patients may not have received care in participating hemophilia treatment centers, and registration, initiated in 1994, largely included prevalent cases. In addition, PWH followed in hemophilia treatment centers may represent patients more engaged in regular care, whereas those less connected to healthcare services — and potentially in poorer health — may be underrepresented. To account for this non-exhaustive coverage, we adjusted the data from FranceCoag population of PWH. Although these factors may have resulted in a slight overestimation of LE, particularly for mild PWH, the FranceCoag registry remains broadly representative of PWH in France (more than 34 treatment centers today), and LE estimates at ages 20 and 40 are likely to provide an accurate reflection of survival patterns. Then, potential biases related to cohort design should be considered. Clinical cohorts may overestimate LE as they include patients engaged in care and may exclude individuals not linked to care or dying early, leading to survivor selection. In contrast, the FranceCoag registry is a national population-based registry designed to capture all PWH, including retrospectively recorded deceased PWH. However, left truncation may have occurred, as deaths before 1994 might not have been fully captured: mild PWH dying before the typical age at diagnosis could have been missed, which could result in a slight overestimation of survival. Given the registry’s national coverage and retrospective data collection, this bias is likely limited. At the same time, a death imputation method was used to address under-ascertainment, however some unrecorded deaths cannot be entirely excluded. To further assess the robustness of our findings, we evaluated the performance of the imputation model used for PWH lost to follow-up. The model demonstrated excellent discrimination (AUC 0.895) and good calibration, with an intercept close to zero and a slope near one, indicating minimal systematic bias and limited overfitting. Overall performance metrics, including a low Brier score and consistent pseudo-R² indices, supported strong predictive accuracy within the study cohort. Sensitivity analyses using a higher assumed mortality rate among PWH lost to follow-up (30% vs. 19% in the primary analysis) resulted in only modest changes in LE estimates. Taken together, these findings indicate that PWH LE estimates are robust to plausible variations in imputation assumptions and are unlikely to be substantially biased by uncertainties related to loss to follow-up. Finally, life tables assume stable mortality rates over time, which may not fully reflect temporal changes in management and outcomes. Furthermore, LE was not stratified by sex due to the small number of women with hemophilia. Cohort life expectancy is theoretically more dynamic, as it accounts for changes in mortality over time. Indeed, it requires near-complete follow-up until cohort extinction and larger sample sizes, which were not available in this relatively small cohort with ongoing inclusions and limited follow-up, particularly among younger PWH.

Further research will enable us to estimate the LE of PWH more exhaustively, notably by matching data from the FranceCoag database with data from the SNDS, and also to study the related quality of life.

Finally, this first French study reveals that people with mild, moderate and severe hemophilia have LE at 20 and 40 years that are comparable to those of the general French population, and higher than those of people living with HIV. In addition, across all severities, remaining LE estimates indicate that PWH now have survival prospects approaching those of the general population, with potential implications for long-term social and financial planning. These findings further support the inclusion of hemophilia in the AERAS grid.

## Supplementary Information

Below is the link to the electronic supplementary material.

Supplementary Material 1
