# Supplementary material for: Life expectancy of people with hemophilia in France in 2022
Source: Eur J Epidemiol. 2026 Apr 22;41(6):743–53. doi: 10.1007/s10654-026-01389-z (PMC13423971; doi:10.1007/s10654-026-01389-z)
Supplement: Supplementary file 1 — Supplementary Material 1 [file 10654_2026_1389_MOESM1_ESM.docx]

**Life expectancy of people with hemophilia in France in 2022.**

**Supplemental Background**

**Hemophilia severity classification**

The hemophilia severity classification is reported by the clinician on the basis of the patient's clinical presentation and the latest biological assays by chronometric technique performed in basal situation (without substitution) prior to the visit:

- clotting factor level < 0.01 IU/ml (<1%) = severe hemophilia
- clotting factor levels between 0.01 and 0.05 IU/ml (1 to 5%) = moderate hemophilia
- clotting factor levels between 0.05 and <0.4 IU/ml (>5% and <40%) = mild hemophilia

**AERAS grid (s'Assurer et Emprunter avec un Risque Aggravé de Santé)**

Access to credit represents a major social issue, particularly for people applying for loans who are considered to be in a situation of aggravated health risk (AHR) due to illness or disability. Indeed, borrower insurance is often a prerequisite for obtaining a loan. For individuals whose illness or disability is deemed an AHR by insurers or banks, moving forward with their plans can be jeopardized if they are unable to obtain insurance coverage under standard contract conditions (i.e., without increased premiums or exclusions of coverage).

A formal agreement between the French government and insurance providers was initiated in 1991 and has continued since then, with the aim of improving access to borrower insurance and credit for people with an AHR.

The AERAS reference grid was established in 2015 as part of the revision of these agreements, to facilitate access to borrower insurance for a number of listed medical conditions (<https://www.aeras-infos.fr/sites/aeras/accueil.html>). This grid was developed by insurance physicians, members of scientific advisory boards, patient advocacy groups, and representatives from government ministries and public expert agencies.

The AERAS Convention is automatically applied by insurance and banking professionals whenever individuals applying for insurance or credit present – or have presented – an AHR , and is updated annually to include additional chronic diseases, provided that therapeutic advances and scientific data demonstrate the ability of relevant treatments to significantly and sustainably reduce the impact of these conditions. The grid specifies, on a condition-by-condition basis, the terms under which individuals may obtain insurance – either without extra premiums or exclusions, or with adjusted premiums and limited coverage.

To date, hemophilia does not appear on the reference grid established under the AERAS Convention, thus this type of insurance is often difficult to obtain – and in some cases entirely denied – for people with hemophilia (PWH), particularly for those with comorbidities such as HIV or HCV: indeed, they face the greatest difficulties in obtaining insurance, with rejection rates reaching around 90%.

In contrast, other conditions that may affect PWH (such as HCV and HIV) – and that insurers might have feared that could reduce LE or lead to work interruptions – are already included in the AERAS reference grid: thus, non-hemophilic individuals living with HIV, can now obtain insurance relatively easily.

Yet, since the late 1990s, not only have treatment strategies significantly improved (such as the eradication of inhibitors through immune tolerance induction and the development of early long-term prophylaxis, including for patients with inhibitors), but also the medications themselves have also continued to evolve.

**Supplemental Methods**

**Correction of a potential underestimation of deaths among those lost to follow-up**

Patients lost to follow-up (LTFU) were defined as those whose last known recorded contact date was more than one, two and four years before the end of the study period for people with severe, moderate and mild hemophilia respectively. To correct for a potential underestimation of deaths among those registered as lost to follow-up, a multivariable logistic regression model was estimated on the population of non-lost to follow-up and lost to follow-up with notified deaths to predict deaths based on their demographic and clinical characteristics (sex, age, type of severity, infections, liver disease excluding infection, onset of inhibitor, severe bleeding, initiation of treatment during the contaminated blood period, time between last FranceCoag visit and today or death, circumstances of diagnosis). This model was then applied to patients lost to follow-up to predict their probability of death. Finally, only the 19% (death rate found in a sample of people lost to follow-up in FranceCoag) of people with the highest probability of death were considered to have died.

The model equation was: P(Death) = $\frac{\text{e}^{\text{β}\text{0+}\text{β}\text{1sex+}\text{β}\text{2age+}\text{β}\text{3severity+}\text{β}\text{4infection+}\text{β}\text{5liverdisease+}\text{β}\text{6inhibitor+}\text{β}\text{7bleeding+}\text{β}\text{8treatmentperiod+}\text{β}\text{9followupperiod+}\text{β}\text{10diagnosiscircumstancies}}}{\text{1+}\text{e}^{\text{β}\text{0+}\text{β}\text{1sex+}\text{β}\text{2age+}\text{β}\text{3severity+}\text{β}\text{4infection+}\text{β}\text{5liverdisease+}\text{β}\text{6inhibitor+}\text{β}\text{7bleeding+}\text{β}\text{8treatmentperiod+}\text{β}\text{9followupperiod+}\text{β}\text{10diagnosiscircumstancies}}}$

To assess the sensitivity of LE estimates to assumptions regarding mortality among PWH LTFU, we conducted additional analyses using higher imputed death proportions. While our primary analysis assumed a 19% mortality rate among LTFU PWH, published estimates range from 9% to 36% depending on the chronic condition. Under a more conservative scenario assuming 30% mortality among LTFU, LE estimates changed only modestly (Supplemental Tables 1 and 2).

| **Age** | **Severe PWH** | **Severe PWH deaths** | **Severe PWH LE** | **Moderate PWH** | **Moderate PWH deaths** | **Moderate PWH LE** | **Mild PWH** | **Mild PWH deaths** | **Mild PWH LE** |
| --- | --- | --- | --- | --- | --- | --- | --- | --- | --- |
| [0,1) | 16 | 8 | 46.28389 | 6 | 1 | 71.85491 | 27 | 0 | 85.35432 |
| [1,5) | 386 | 4 | 75.80648 | 87 | 0 | 83.82853 | 251 | 2 | 84.35432 |
| [5,10) | 1,949 | 1 | 74.93056 | 528 | 0 | 79.82853 | 1,298 | 2 | 83.02167 |
| [10,15) | 4,121 | 1 | 70.11661 | 1,315 | 0 | 74.82853 | 3,755 | 1 | 78.64442 |
| [15,20) | 5,467 | 4 | 65.19870 | 1,949 | 0 | 69.82853 | 7,088 | 2 | 73.74588 |
| [20,25) | 6,565 | 11 | 60.42849 | 2,271 | 4 | 64.82853 | 6,806 | 2 | 68.84647 |
| [25,30) | 7,505 | 9 | 55.91585 | 3,916 | 0 | 60.37986 | 13,395 | 2 | 63.94402 |
| [30,35) | 9,164 | 20 | 51.23709 | 4,176 | 3 | 55.37986 | 14,483 | 8 | 58.98991 |
| [35,40) | 8,871 | 38 | 46.77184 | 4,976 | 7 | 50.57015 | 17,847 | 7 | 54.14614 |
| [40,45) | 7,254 | 34 | 42.73032 | 4,321 | 20 | 45.90946 | 19,284 | 16 | 49.24753 |
| [45,50) | 5,928 | 48 | 38.68431 | 4,526 | 22 | 41.92583 | 16,523 | 42 | 44.44186 |
| [50,55) | 7,181 | 71 | 35.17953 | 5,555 | 21 | 37.89583 | 16,699 | 31 | 39.97833 |
| [55,60) | 6,657 | 64 | 31.83603 | 4,156 | 24 | 33.57126 | 23,430 | 66 | 35.32783 |
| [60,65) | 5,285 | 45 | 28.28093 | 5,294 | 34 | 29.48155 | 23,210 | 83 | 30.79347 |
| [65,70) | 3,565 | 34 | 24.40238 | 3,582 | 24 | 25.36211 | 22,826 | 80 | 26.30393 |
| [70,75) | 2,311 | 24 | 20.47232 | 3,698 | 24 | 21.14106 | 22,206 | 79 | 21.72475 |
| [75,80) | 1,085 | 11 | 16.43042 | 3,449 | 18 | 16.75594 | 17,126 | 53 | 17.06979 |
| [80,85) | 712 | 5 | 12.15493 | 2,139 | 16 | 12.13286 | 14,628 | 60 | 12.29700 |
| [85,120] | 310 | 3 | 7.50000 | 1,178 | 22 | 7.50000 | 14,263 | 70 | 7.50000 |

**Supplemental Table 1: Life expectancy with a threshold of 19% mortality rate among patients lost to follow-up**

PWH: People With Hemophilia; LE: Life Expectancies

| **Age** | **Severe PWH** | **Severe PWH deaths** | **Severe PWH LE** | **Moderate PWH** | **Moderate PWH deaths** | **Moderate PWH LE** | **Mild PWH** | **Mild PWH deaths** | **Mild PWH LE** |
| --- | --- | --- | --- | --- | --- | --- | --- | --- | --- |
| [0,1) | 16 | 9 | 44.58487 | 6 | 1 | 71.80202 | 27 | 0 | 84.46015 |
| [1,5) | 386 | 4 | 78.08607 | 87 | 0 | 83.76603 | 251 | 2 | 83.46015 |
| [5,10) | 1,949 | 1 | 77.30664 | 528 | 0 | 79.76603 | 1,298 | 2 | 82.09853 |
| [10,15) | 4,080 | 1 | 72.49880 | 1,315 | 0 | 74.76603 | 3,755 | 1 | 77.71415 |
| [15,20) | 5,302 | 0 | 67.58464 | 1,949 | 0 | 69.76603 | 7,064 | 2 | 72.81436 |
| [20,25) | 6,239 | 4 | 62.58464 | 2,169 | 1 | 64.76603 | 6,772 | 3 | 67.91397 |
| [25,30) | 7,181 | 6 | 57.77755 | 3,879 | 1 | 59.90973 | 13,161 | 2 | 63.05903 |
| [30,35) | 8,097 | 7 | 53.00897 | 3,691 | 5 | 54.98378 | 13,956 | 4 | 58.10506 |
| [35,40) | 5,020 | 9 | 48.22777 | 3,477 | 1 | 50.34047 | 16,737 | 6 | 53.18480 |
| [40,45) | 1,352 | 5 | 43.63953 | 2,106 | 6 | 45.40931 | 16,718 | 7 | 48.27573 |
| [45,50) | 942 | 10 | 39.40734 | 1,957 | 13 | 41.02494 | 13,820 | 59 | 43.37167 |
| [50,55) | 714 | 6 | 36.41974 | 2,157 | 14 | 37.32612 | 13,704 | 71 | 39.25352 |
| [55,60) | 461 | 4 | 32.87552 | 1,576 | 12 | 33.47496 | 19,095 | 70 | 35.21811 |
| [60,65) | 947 | 8 | 29.22255 | 2,102 | 13 | 29.67709 | 19,725 | 69 | 30.82336 |
| [65,70) | 327 | 2 | 25.37563 | 1,548 | 10 | 25.53068 | 19,878 | 73 | 26.32312 |
| [70,75) | 302 | 3 | 21.08605 | 1,719 | 8 | 21.28678 | 18,924 | 62 | 21.76461 |
| [75,80) | 158 | 1 | 17.03271 | 1,605 | 9 | 16.72908 | 13,950 | 40 | 17.08280 |
| [80,85) | 54 | 0 | 12.50000 | 938 | 7 | 12.13370 | 12,214 | 51 | 12.29338 |
| [85,120] | 92 | 1 | 7.50000 | 684 | 13 | 7.50000 | 11,690 | 56 | 7.50000 |

**Supplemental Table 2: Life expectancy with a threshold of 30% mortality rate among patients lost to follow-up**

PWH: People With Hemophilia; LE: Life Expectancies

Differences were ≤1 year among mild PWH (at birth, 20, and 40 years), no change was observed among those with moderate disease, and variations ranged from −2 years at birth to +2 years at age 20 and +1 year at age 40 among severe PWH. Overall, these findings indicate that LE estimates were relatively robust to plausible variations in mortality assumptions among LTFU PWH.

Supplemental Table 3 presents the model used to estimate the factors associated with deaths among PWH who were not LTFU and those who were LTFU with reported deaths, thereby allowing the probability of death to be deduced for each PWH LTFU for whom no death had been previously reported.

|  | **Estimate** | **Std. Error** | **t value** | **Pr(>\|t\|)** |  |
| --- | --- | --- | --- | --- | --- |
| (Intercept) | -2.79966 | 0.54011 | -5.183 | 2.24E-07 | *** |
| age | 0.30125 | 0.01452 | 20.744 | < 2e-16 | *** |
| sex | -1.84943 | 0.46589 | -3.97 | 7.27E-05 | *** |
| type of severity: severe | - | - | - | - |  |
| type of severity: moderate | -1.44917 | 0.19662 | -7.371 | 1.90E-13 | *** |
| type of severity: mild | -1.66327 | 0.19402 | -8.572 | < 2e-16 | *** |
| prophylactic treatment | -1.99022 | 0.18097 | -10.998 | < 2e-16 | *** |
| onset of inhibitor, severe bleeding | 0.50739 | 0.16148 | 3.142 | 0.00168 | ** |
| severe bleeding | 1.36193 | 0.13665 | 9.966 | < 2e-16 | *** |
| infections | 0.93883 | 0.11012 | 8.526 | < 2e-16 | *** |
| initiation of treatment during the contaminated blood period | -0.17665 | 0.12207 | -1.447 | 0.14792 |  |
| liver disease excluding infection | 1.95258 | 0.30912 | 6.317 | 2.84E-10 | *** |
| time between last FranceCoag visit and today or death | -0.01098 | 0.01789 | -0.614 | 0.53939 |  |
| circumstances of diagnosis: family history | - | - | - | - |  |
| circumstances of diagnosis: bleeding | 0.0663 | 0.12838 | 0.516 | 0.60555 |  |
| circumstances of diagnosis: blood test | 0.26936 | 0.15156 | 1.777 | 0.07558 | . |
| circumstances of diagnosis: unknown | 0.18702 | 0.19935 | 0.938 | 0.34821 |  |

**Supplemental Table 3: Multivariable logistic regression model of factors associated with deaths among PWH who were not lost to follow-up or lost to follow-up with reported deaths**

Several performance measures were carried out on this imputation model for patients lost to follow-up:

- ROC curve (Supplemental Figure I): The model shows very good discrimination (AUC = 0.895; 95% CI 0.883–0.907), reflecting a good ability to differentiate between deceased and non-deceased subjects.
- Calibration diagram (Supplemental Figure II): The calibration curve shows good agreement between the probabilities predicted by the model and the observed probabilities. The logistic calibration curve is very close to the ideal diagonal, indicating satisfactory fit across the entire risk spectrum. The calibration intercept (−0.066) is close to zero, suggesting the absence of major systematic bias in over- or underestimation. The calibration slope (0.967) is close to 1, reflecting limited overfitting and good model stability. Calibration deviations are small (Emax = 0.036; Eavg = 0.005), and the Brier score (0.062) confirms good overall performance. Discrimination is high (C-statistic = 0.895), indicating excellent ability to distinguish subjects with and without the event. Overall, these results suggest that the model demonstrates adequate calibration and satisfactory predictive performance within the study cohort.
- Performance indicators (Supplemental Table 4): The pseudo-R² measures confirm the good explanatory power of the model:
- McFadden’s R² is 0.382, a value considered good for logistic regression, reflecting an improvement over the null model.
- Cox & Snell R² is 0.23 and Nagelkerke R² is 0.464, indicating that approximately 46% of the theoretically maximum explainable variability is captured by the model.
- Tjur’s R² (0.314) shows an average separation of 31% between predicted probabilities for deceased and non-deceased subjects, confirming good discrimination, consistent with the observed AUC (0.895).

Overall, these indicators suggest that the model demonstrates robust explanatory and discriminative performance within the study cohort.


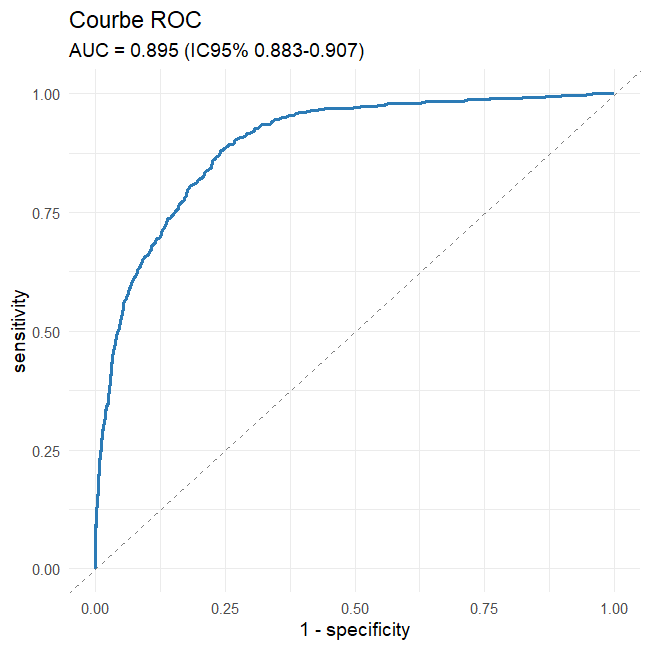


**Supplemental Figure I: ROC curve of the imputation model**

**Supplemental Figure II: Calibration diagram of the imputation model**

| R2_McFadden | 0.382 |
| --- | --- |
| Cox & Snell R² | 0.23 |
| Nagelkerke R² | 0.464 |
| Tjur R² | 0.314 |

**Supplemental Table 4: Performance indicators of the imputation model**

**Hemophilia population Adjustment Method**

The FranceCoag database provides an exhaustive record of the active file for severe and moderate hemophilia. However, some centers in France do not participate in FranceCoag: the total PWH active file is therefore potentially reduced. At the same time, the registration of mild PWH is not exhaustive in FranceCoag, as they are not mainly managed in reference centers, which leads to a low representativeness of mild PWH. A technique for adjusting data by severity type and age category was applied, using 2021 data from the Canadian Hemophilia Registry (CHR), one of the largest and most exhaustive international registries in the field, as a theoretical data source to take account of the non-representativeness of the FranceCoag registry for hemophilia, particularly mild hemophilia. Weighting coefficients were calculated and used to adjust the numbers of PWH in the FranceCoag registry by age group and severity. The coefficients obtained are presented in Supplemental Table 5.

| **A/Severe hemophilia** | | | | | |
| --- | --- | --- | --- | --- | --- |
| AGE | FranceCoag’s hemophilia A or B | Canada’s hemophilia A | Canada’s hemophilia B | Canada’s hemophilia A or B | Coefficients |
| [0,4] | 199 (7.0%) | 52 | 11 | 63 (4.9%) | 0.70 |
| [5,9] | 206 (7.2%) | 104 | 13 | 117 (9.0%) | 1.25 |
| [10,14] | 235 (8.2%) | 122 | 25 | 147 (11.4%) | 1.39 |
| [15,24] | 469 (16.0%) | 236 | 30 | 266 (20.5%) | 1.28 |
| [25,34] | 481 (17.0%) | 223 | 31 | 254 (19.6%) | 1.15 |
| [35,44] | 451 (16.0%) | 164 | 27 | 191 (14.7%) | 0.91 |
| [45,54] | 383 (13.0%) | 87 | 29 | 116 (8.9%) | 0.68 |
| [55,64] | 254 (8.9%) | 65 | 27 | 92 (7.1%) | 0.79 |
| [65,74] | 142 (5.0%) | 31 | 8 | 39 (3.0%) | 0.60 |
| [75,84] | 34 (1.2%) | 9 | 2 | 11 (0.8%) | 0.67 |
| [85,100] | 7 (0.2%) | 1 | 0 | 1 (0.1%) | 0.50 |
| Total | 2861 | 1094 | 203 | 1297 |  |
| **B/Moderate hemophilia** | | | | | |
| AGE | FranceCoag’s hemophilia A or B | Canada’s hemophilia A | Canada’s hemophilia B | Canada’s hemophilia A or B | Coefficients |
| [0,4] | 81 (5.5%) | 8 | 4 | 12 (2.1%) | 0.38 |
| [5,9] | 103 (7.0%) | 19 | 7 | 26 (4.5%) | 0.64 |
| [10,14] | 111 (7.5%) | 25 | 14 | 39 (6.9%) | 0.92 |
| [15,24] | 245 (17%) | 51 | 31 | 82 (14.5%) | 0.85 |
| [25,34] | 203 (14%) | 49 | 54 | 103 (18.2%) | 1.30 |
| [35,44] | 201 (14%) | 49 | 44 | 93 (16.4%) | 1.17 |
| [45,54] | 163 (11%) | 40 | 37 | 77 (13.6%) | 1.23 |
| [55,64] | 155 (11%) | 38 | 25 | 63 (11.1%) | 1.01 |
| [65,74] | 114 (7.7%) | 17 | 23 | 40 (7.0%) | 0.91 |
| [75,84] | 68 (4.6%) | 14 | 13 | 27 (4.8%) | 1.04 |
| [85,100] | 28 (1.9%) | 1 | 4 | 5 (0.9%) | 0.47 |
| Total | 1472 | 311 | 256 | 567 |  |
| **C/Mild hemophilia** | | | | | |
| AGE | FranceCoag’s hemophilia A or B | Canada’s hemophilia A | Canada’s hemophilia B | Canada’s hemophilia A or B | Coefficients |
| [0,4] | 255 (4.5%) | 25 | 5 | 30 (1.5%) | 0.33 |
| [5,9] | 394 (7.0%) | 51 | 7 | 58 (2.8%) | 0.40 |
| [10,14] | 472 (8.4%) | 92 | 11 | 103 (5.0%) | 0.60 |
| [15,24] | 1,070 (19%) | 217 | 29 | 246 (11.9%) | 0.63 |
| [25,34] | 816 (14%) | 275 | 47 | 322 (15.6%) | 1.11 |
| [35,44] | 684 (12%) | 297 | 33 | 330 (16.0%) | 1.33 |
| [45,54] | 593 (11%) | 223 | 30 | 253 (12.2%) | 1.11 |
| [55,64] | 528 (9.4%) | 256 | 27 | 283 (13.7%) | 1.46 |
| [65,74] | 455 (8.1%) | 211 | 25 | 236 (11.4%) | 1.41 |
| [75,84] | 272 (4.8%) | 118 | 27 | 145 (7.0%) | 1.46 |
| [85,100] | 94 (1.7%) | 55 | 4 | 59 (2.9%) | 1.71 |
| Total | 5634 | 1820 | 245 | 2065 |  |

**Supplemental Table 5: Calculation of weighting coefficients from the Canadian registry of PWH and the FranceCoag registry.**

PWH: People With Hemophilia

**Life expectancy estimates - Abbreviated mortality tables**

Deaths of individuals by five-year age category were divided by the exposed population to calculate mortality rates and estimate abridged life tables and life expectancies (LE). The duration of exposure was the period between the date of birth and the date of the last news item. The latter corresponded to the date of death or the date of the last visit to the hemophilia center, except for patients lost to follow-up who may have died, in which case it was calculated as the sum of the exposure time from birth to the last visit and the median time to death since the last live visit for those not lost to follow-up and declared dead (calculated for deaths observed in the sample and stratified by severity).

Due to the relatively low proportion of participants in the older categories, the last age category constructed in the mortality tables was 85 years and over for people with mild and moderate hemophilia, and 80 years and over for people with severe hemophilia. The corresponding confidence intervals were obtained using bootstrap resampling techniques. One thousand bootstrap simulations were carried out for each sub-population; the 2.5th and 97.5th percentiles of these observations were taken as the limits for the 95% bootstrap confidence interval.

**Life expectancy comparison**

For comparisons, p-values (tests of statistical significance) were calculated using bootstrap resampling techniques. For this, bootstrap samples were generated assuming the null hypothesis, then examined according to the frequency with which the simulated data were as extreme, or more extreme, than the observed data. Thus, if the p-value was below the α threshold at 5%, the null hypothesis was rejected and the observed difference between the values was considered statistically significant. Comparisons with LE data for the general population or for HIV patients were made against a reference value. Comparisons of LE between two groups of hemophilia patients of different severity were made by comparing the differences between their respective values to zero.

**Supplemental Results - Imputation of deaths and adjustment of numbers of PWH**

Supplemental Table 6 shows the model used to estimate the factors associated with death for participants not lost to follow-up and those lost to follow-up with notified death, enabling us to deduce the probability of death for each of the participants lost to follow-up for whom no death had been notified previously.

| Variables | β | OR | p-value |
| --- | --- | --- | --- |
| **Intercept** | **-2.8** |  | **<0.001** |
| **Age at last visit (in years)** | 0.3 | 1.4 | **<0.001** |
| **Sex** |  |  |  |
| Female | -1.8 | 0.2 | **<0.001** |
| Male | 0 | 1 | - |
| **Severity** |  |  |  |
| Mild | -1.7 | 0.2 | **<0.001** |
| Moderate | -1.4 | 0.2 | **<0.001** |
| Severe | 0 | 1 | - |
| **Infection at inclusion or during follow-up (HIV, HCV, HBV)** |  |  |  |
| Yes | 0.94 | 2.6 | **<0.001** |
| No | 0 | 1 | - |
| **Liver disease other than infection at inclusion/during follow-up** |  |  |  |
| Yes | 2.0 | 7.1 | **<0.001** |
| No | 0 | 1 | - |
| **Severe bleeding during follow-up** |  |  |  |
| Yes | 1.4 | 3.9 | **<0.001** |
| No | 0 | 1 | - |
| **Occurrence of inhibitors at inclusion or during follow-up** |  |  |  |
| Yes | 0.5 | 1.7 | **<0.002** |
| No | 0 | 1 | - |
| **Prophylaxis during follow-up** |  |  |  |
| Yes | -2.0 | 0.1 | **<0.001** |
| No | 0 | 1 | - |
| **Initiation of replacement therapy during period at risk of transmission of blood-borne viruses** |  |  |  |
| <=1990 | -0.2 | 0.8 | 0.150 |
| >1990 | 0 | 1 | - |
| **Time between last visit and now or death (in years)** | -0.01 | 1.0 | 0.500 |
| **Diagnosis circumstances** |  |  |  |
| Secondary to a bleeding event | 0.1 | 1.1 | 0.600 |
| Fortuitously during a routine hemostasis test | 0.3 | 1.3 | 0.076 |
| Unknown | 0.2 | 1.2 | 0.300 |
| Screening due to family history | 0 | 1 | - |

**Supplemental Table 6:** **Multivariable logistic regression model of factors associated with death in PWH not lost to follow-up or lost to follow-up with reported death. (FranceCoag, n= 7,038)**

OR: Odds Ratio; β: Regression coefficient; PWH: People With Hemophilia; HIV: Human immunodeficiency virus; HCV: hepatitis C virus; HBV: hepatitis B virus.

Subsequently, model-based probabilities of death were calculated for participants lost to follow-up for whom there was no information on whether they were dead or alive. The probabilities were then arranged in descending order, and the 19% of lost-of-sight participants with the highest probabilities of death were classified as deceased. The flowchart describing the steps involved in selecting the overall potential deaths is shown in Supplemental Figure III.

Total calculated deaths

n = 1,282

**Supplemental Figure III: Steps for selecting total calculated deaths in PWH**

PWH: People With Hemophilia

Total calculated deaths, including observed and theoretical deaths, were therefore 1282 in the hemophilia population included in the study.

**Supplemental Results -** **Mortality among PWH**

Using the crude numbers and weighting coefficients obtained from the Canadian registry, weighted numbers of PWH followed-up in FranceCoag were calculated in general and by severity. These numbers are presented in Supplemental Tables 7, 7a, 7b and 7c. Similarly, the total number of deaths calculated by age group, after estimating 19% mortality among participants lost to follow-up with the highest probability of death, is presented in Supplemental Table 7.

| Age groups (in years) | Number of patients | Weighted number of patients | Weighted number PY of follow-up | Number of potential deaths | Number of calculated deaths | Specific mortality rate (for 1000 PY)* |
| --- | --- | --- | --- | --- | --- | --- |
| [0-1) | 97 | 45 | 49 | 9 | 6 | 122.4 |
| [1-5) | 438 | 209 | 724 | 6 | 4 | 5.5 |
| [5-10) | 702 | 481 | 3775 | 3 | 2 | 0.5 |
| [10-15) | 814 | 706 | 9191 | 2 | 2 | 0.2 |
| [15-20) | 975 | 798 | 14504 | 6 | 6 | 0.4 |
| [20-25) | 799 | 675 | 15642 | 17 | 18 | 1.2 |
| [25-30) | 763 | 877 | 24816 | 11 | 12 | 0.5 |
| [30-35) | 728 | 836 | 27823 | 31 | 36 | 1.3 |
| [35-40) | 718 | 829 | 31694 | 52 | 52 | 1.6 |
| [40-45) | 607 | 715 | 30859 | 70 | 75 | 2.4 |
| [45-50) | 567 | 560 | 26977 | 112 | 107 | 4.0 |
| [50-55) | 569 | 556 | 29435 | 123 | 108 | 3.7 |
| [55-60) | 493 | 590 | 34243 | 154 | 171 | 5.0 |
| [60-65) | 442 | 537 | 33789 | 162 | 191 | 5.7 |
| [65-70) | 384 | 442 | 29973 | 138 | 155 | 5.2 |
| [70-75) | 325 | 388 | 28215 | 127 | 147 | 5.2 |
| [75-80) | 215 | 279 | 21660 | 82 | 103 | 4.8 |
| [80-85) | 159 | 212 | 17479 | 81 | 108 | 6.2 |
| [85-120] | 129 | 178 | 15751 | 95 | 132 | 8.4 |
| Total | 9924 | 9913 | 396599 | 1281 | 1435 | 3.6 |

**Supplemental Table 7: Numbers and deaths of FranceCoag PWH weighted on the basis of PWH in the Canadian registry**

PY: Person-year of follow-up; *Specific mortality rate = (Weighted deaths*1000)/ Weighted PY of follow-up

| Age groups (in years) | Number of patients | Weighted number of patients | Weighted number PY of follow-up | Number of potential deaths | Number of calculated deaths | Specific mortality rate (for 1000 PY)* |
| --- | --- | --- | --- | --- | --- | --- |
| [0-1) | 33 | 23 | 16 | 8 | 6 | 500.0 |
| [1-5) | 166 | 116 | 386 | 4 | 3 | 10.4 |
| [5-10) | 206 | 258 | 1949 | 1 | 1 | 0.5 |
| [10-15) | 232 | 322 | 4121 | 1 | 1 | 0.2 |
| [15-20) | 241 | 308 | 5467 | 4 | 5 | 0.7 |
| [20-25) | 225 | 288 | 6565 | 11 | 14 | 1.7 |
| [25-30) | 234 | 269 | 7505 | 9 | 10 | 1.2 |
| [30-35) | 243 | 279 | 9164 | 20 | 23 | 2.2 |
| [35-40) | 258 | 235 | 8871 | 38 | 35 | 4.3 |
| [40-45) | 187 | 170 | 7254 | 34 | 31 | 4.7 |
| [45-50) | 182 | 124 | 5928 | 48 | 33 | 8.1 |
| [50-55) | 201 | 137 | 7181 | 71 | 48 | 9.9 |
| [55-60) | 146 | 115 | 6657 | 64 | 51 | 9.6 |
| [60-65) | 107 | 85 | 5285 | 45 | 36 | 8.5 |
| [65-70) | 88 | 53 | 3565 | 34 | 20 | 9.5 |
| [70-75) | 53 | 32 | 2311 | 24 | 14 | 10.4 |
| [75-80) | 21 | 14 | 1085 | 11 | 7 | 10.1 |
| [80-85) | 13 | 9 | 712 | 5 | 3 | 7.0 |
| [85-120] | 7 | 4 | 310 | 3 | 2 | 9.7 |
| Total | 2843 | 2841 | 84332 | 435 | 343 | 5.2 |

**Supplemental Table 7a: Numbers and deaths of severe hemophilia patients in FranceCoag weighted on the basis of hemophilia patients in the Canadian registry**

PY: Person-year of follow-up; *Specific mortality rate = (Weighted deaths*1000)/ Weighted PY of follow-up

| Age groups (in years) | Number of patients | Weighted number of patients | Weighted number PY of follow-up | Number of potential deaths | Number of calculated deaths | Specific mortality rate (for 1000 PY)* |
| --- | --- | --- | --- | --- | --- | --- |
| [0-1) | 16 | 6 | 6 | 1 | 0 | 166.7 |
| [1-5) | 65 | 25 | 87 | 0 | 0 | 0.0 |
| [5-10) | 103 | 66 | 528 | 0 | 0 | 0.0 |
| [10-15) | 110 | 101 | 1315 | 0 | 0 | 0.0 |
| [15-20) | 127 | 108 | 1949 | 0 | 0 | 0.0 |
| [20-25) | 115 | 98 | 2271 | 4 | 3 | 1.8 |
| [25-30) | 106 | 138 | 3916 | 0 | 0 | 0.0 |
| [30-35) | 96 | 125 | 4176 | 3 | 4 | 0.7 |
| [35-40) | 111 | 130 | 4976 | 7 | 8 | 1.4 |
| [40-45) | 86 | 101 | 4321 | 20 | 23 | 4.6 |
| [45-50) | 76 | 93 | 4526 | 22 | 27 | 4.9 |
| [50-55) | 85 | 105 | 5555 | 21 | 26 | 3.8 |
| [55-60) | 71 | 72 | 4156 | 24 | 24 | 5.8 |
| [60-65) | 83 | 84 | 5294 | 34 | 34 | 6.4 |
| [65-70) | 58 | 53 | 3582 | 24 | 22 | 6.7 |
| [70-75) | 56 | 51 | 3698 | 24 | 22 | 6.5 |
| [75-80) | 43 | 45 | 3449 | 18 | 19 | 5.2 |
| [80-85) | 25 | 26 | 2139 | 16 | 17 | 7.5 |
| [85-120] | 28 | 13 | 1178 | 22 | 10 | 18.7 |
| Total | 1460 | 1440 | 57122 | 240 | 239 | 4.2 |

**Supplemental Table 7b: Numbers and deaths of moderate hemophilia patients in FranceCoag weighted on the basis of hemophilia patients in the Canadian registry**

PY: Person-year of follow-up; *Specific mortality rate = (Weighted deaths*1000)/ Weighted PY of follow-up

| Age groups (in years) | Number of patients | Weighted number of patients | Weighted number PY of follow-up | Number of potential deaths | Number of calculated deaths | Specific mortality rate (for 1000 PY)* |
| --- | --- | --- | --- | --- | --- | --- |
| [0-1) | 48 | 16 | 27 | 0 | 0 | 0.0 |
| [1-5) | 207 | 68 | 251 | 2 | 1 | 8.0 |
| [5-10) | 393 | 157 | 1298 | 2 | 1 | 1.5 |
| [10-15) | 472 | 283 | 3755 | 1 | 1 | 0.3 |
| [15-20) | 607 | 382 | 7088 | 2 | 1 | 0.3 |
| [20-25) | 459 | 289 | 6806 | 2 | 1 | 0.3 |
| [25-30) | 423 | 470 | 13395 | 2 | 2 | 0.1 |
| [30-35) | 389 | 432 | 14483 | 8 | 9 | 0.6 |
| [35-40) | 349 | 464 | 17847 | 7 | 9 | 0.4 |
| [40-45) | 334 | 444 | 19284 | 16 | 21 | 0.8 |
| [45-50) | 309 | 343 | 16523 | 42 | 47 | 2.5 |
| [50-55) | 283 | 314 | 16699 | 31 | 34 | 1.9 |
| [55-60) | 276 | 403 | 2343 | 66 | 96 | 28.2 |
| [60-65) | 252 | 368 | 2321 | 83 | 121 | 35.8 |
| [65-70) | 238 | 336 | 22826 | 80 | 113 | 3.5 |
| [70-75) | 216 | 305 | 22206 | 79 | 111 | 3.6 |
| [75-80) | 151 | 220 | 17126 | 53 | 77 | 3.1 |
| [80-85) | 121 | 177 | 14628 | 60 | 88 | 4.1 |
| [85-120] | 94 | 161 | 14263 | 70 | 120 | 4.9 |
| Total | 5621 | 5632 | 213169 | 606 | 853 | 2.8 |

**Supplemental Table 7c: Numbers and deaths of mild hemophilia patients in FranceCoag weighted on the basis of hemophilia patients in the Canadian registry**

PY: Person-year of follow-up; *Specific mortality rate = (Weighted deaths*1000)/ Weighted PY of follow-up

The mortality rates calculated for each severity from the weighted numbers and calculated deaths and used in the abbreviated mortality tables are shown in Supplemental Table 8.

|  | Severe hemophilia | | | Moderate hemophilia | | | Mild hemophilia | | |
| --- | --- | --- | --- | --- | --- | --- | --- | --- | --- |
| **Age groups (in years)** | **Weighted number PY of follow-up** | **Number of deaths** | **Q_(x)_ Specific mortality rate (for 1000 PY)** | **Weighted number PY of follow-up** | **Number of deaths** | **Q_(x)_ Specific mortality rate (for 1000 PY)** | **Weighted number PY of follow-up** | **Number of deaths** | **Q_(x)_ Specific mortality rate (for 1000 PY)** |
| [0,1) | 16 | 8 | 500.0 | 6 | 1 | 166.7 | 27 | 0 | 0.0 |
| [1,5) | 386 | 4 | 10.4 | 87 | 0 | 0.0 | 251 | 2 | 8.0 |
| [5,10) | 1949 | 1 | 0.5 | 528 | 0 | 0.0 | 1298 | 2 | 1.5 |
| [10,15) | 4121 | 1 | 0.2 | 1315 | 0 | 0.0 | 3755 | 1 | 0.3 |
| [15,20) | 5467 | 4 | 0.7 | 1949 | 0 | 0.0 | 7088 | 2 | 0.3 |
| [20,25) | 6565 | 11 | 1.7 | 2271 | 4 | 1.8 | 6806 | 2 | 0.3 |
| [25,30) | 7505 | 9 | 1.2 | 3916 | 0 | 0.0 | 13395 | 2 | 0.1 |
| [30,35) | 9164 | 20 | 2.2 | 4176 | 3 | 0.7 | 14483 | 8 | 0.6 |
| [35,40) | 8871 | 38 | 4.3 | 4976 | 7 | 1.4 | 17847 | 7 | 0.4 |
| [40,45) | 7254 | 34 | 4.7 | 4321 | 20 | 4.6 | 19284 | 16 | 0.8 |
| [45,50) | 5928 | 48 | 8.1 | 4526 | 22 | 4.9 | 16523 | 42 | 2.5 |
| [50,55) | 7181 | 71 | 9.9 | 5555 | 21 | 3.8 | 16699 | 31 | 1.9 |
| [55,60) | 6657 | 64 | 9.6 | 4156 | 24 | 5.8 | 23430 | 66 | 28.2 |
| [60,65) | 5285 | 45 | 8.5 | 5294 | 34 | 6.4 | 23210 | 83 | 35.8 |
| [65,70) | 3565 | 34 | 9.5 | 3582 | 24 | 6.7 | 22826 | 80 | 3.5 |
| [70,75) | 2311 | 24 | 10.4 | 3698 | 24 | 6.5 | 22206 | 79 | 3.6 |
| [75,80) | 1085 | 11 | 10.1 | 3449 | 18 | 5.2 | 17126 | 53 | 3.1 |
| [80,85) | 712 | 5 | 7.0 | 2139 | 16 | 7.5 | 14628 | 60 | 4.1 |
| [85,120] | 310 | 3 | 9.7 | 1178 | 22 | 18.7 | 14263 | 70 | 4.9 |
| Total | 84332 | 435 | 5.2 | 57122 | 240 | 4.2 | 213169 | 606 | 2.8 |

**Supplemental Table 8: Age-specific mortality rates stratified by severity in PWH**

PY: Person-year of follow-up

At less than one year, people with severe and moderate hemophilia had the highest mortality rates, at 500.0 and 166.7 deaths per 1000 PYs respectively.

**Supplemental Results – Life expectancies**

LE for each age period are detailed in Supplemental Table 9.

| **Age groups**  **(in years)** | **Total PWH LE** | **Total PWH IC95% LB** | **Total PWH IC95% UB** | **Severe PWH LE** | **Severe PWH IC95% LB** | **Severe PWH IC95% UB** | **Moderate PWH LE** | **Moderate PWH IC95% LB** | **Moderate PWH IC95% UB** | **Mild PWH LE** | **Mild PWH IC95% LB** | **Mild PWH IC95% UB** |
| --- | --- | --- | --- | --- | --- | --- | --- | --- | --- | --- | --- | --- |
| [0,1) | 69.17 | 59.08 | 76.82 | 46.28 | 27.51 | 64.09 | 71.85 | 33.13 | 85.53 | 85.35 | 81.08 | 88.48 |
| [1,5) | 82.05 | 79.56 | 83.97 | 75.81 | 72.76 | 78.46 | 83.83 | 82.91 | 84.73 | 84.35 | 80.08 | 87.48 |
| [5,10) | 80.75 | 80.22 | 81.20 | 74.93 | 74.03 | 75.91 | 79.83 | 78.91 | 80.73 | 83.02 | 82.00 | 83.79 |
| [10,15) | 76.06 | 75.70 | 76.45 | 70.12 | 69.26 | 71.02 | 74.83 | 73.91 | 75.73 | 78.64 | 78.24 | 79.02 |
| [15,20) | 71.14 | 70.80 | 71.50 | 65.20 | 64.36 | 66.05 | 69.83 | 68.91 | 70.73 | 73.75 | 73.38 | 74.08 |
| [20,25) | 66.29 | 65.96 | 66.61 | 60.43 | 59.64 | 61.26 | 64.83 | 63.91 | 65.73 | 68.85 | 68.51 | 69.15 |
| [25,30) | 61.63 | 61.34 | 61.93 | 55.92 | 55.07 | 56.76 | 60.38 | 59.64 | 61.09 | 63.94 | 63.63 | 64.24 |
| [30,35) | 56.77 | 56.47 | 57.60 | 51.24 | 50.39 | 52.05 | 55.38 | 54.64 | 56.09 | 58.99 | 58.70 | 59.27 |
| [35,40) | 52.07 | 51.80 | 52.34 | 46.77 | 45.94 | 47.54 | 50.57 | 49.91 | 51.31 | 54.15 | 53.88 | 54.42 |
| [40,45) | 47.48 | 47.22 | 47.73 | 42.73 | 41.95 | 43.43 | 45.91 | 45.23 | 46.61 | 49.25 | 49.00 | 49.51 |
| [45,50) | 42.99 | 42.75 | 43.23 | 38.68 | 37.94 | 39.35 | 41.93 | 41.36 | 42.54 | 44.44 | 44.21 | 44.69 |
| [50,55) | 38.84 | 38.64 | 39.05 | 35.18 | 34.53 | 35.81 | 37.90 | 37.38 | 38.44 | 39.98 | 39.79 | 40.18 |
| [55,60) | 34.61 | 34.44 | 34.76 | 31.84 | 31.23 | 32.39 | 33.57 | 33.11 | 34.04 | 35.33 | 35.17 | 35.49 |
| [60,65) | 30.34 | 30.19 | 30.46 | 28.28 | 27.73 | 28.84 | 29.48 | 29.11 | 29.85 | 30.79 | 30.66 | 30.94 |
| [65,70) | 26.01 | 25.91 | 26.12 | 24.40 | 23.85 | 24.86 | 25.36 | 25.05 | 25.66 | 26.30 | 26.20 | 26.42 |
| [70,75) | 21.56 | 21.48 | 21.65 | 20.47 | 20.01 | 20.93 | 21.14 | 20.90 | 21.40 | 21.72 | 21.65 | 21.82 |
| [75,80) | 16.99 | 16.94 | 17.05 | 16.43 | 16.06 | 16.81 | 16.76 | 16.59 | 16.93 | 17.07 | 17.01 | 17.14 |
| [80,85) | 12.27 | 12.24 | 12.30 | 12.15 | 11.91 | 12.40 | 12.13 | 12.04 | 12.23 | 12.30 | 12.26 | 12.33 |
| [85,120] | 7.50 | NA | NA | 7.50 | NA | NA | 7.50 | NA | NA | 7.50 | NA | NA |

**Supplemental Table 9: LE (in years) at all ages for total PWH and by severity**

PWH: People With Hemophilia; LE: Life Expectancies; LB: Lower bounds; UB: Upper bounds.

Comparisons between PWH and general population LE are presented for each age period in Supplemental Table 10.

| **Age groups**  **(in years)** | **General population LE** | **Total PWH p-value** | **Severe PWH p-value** | **Moderate PWH p-value** | **Mild PWH p-value** |
| --- | --- | --- | --- | --- | --- |
| [0,1) | 79.3 | 0.016 | 0.002 | 0.652 | 0.018 |
| [1,5) | 78.6 | 0.014 | 0.038 | < 0.001 | 0.02 |
| [5,10) | 74.7 | < 0.001 | 0.6 | < 0.001 | < 0.001 |
| [10,15) | 69.7 | < 0.001 | 0.336 | < 0.001 | < 0.001 |
| [15,20) | 64.8 | < 0.001 | 0.358 | < 0.001 | < 0.001 |
| [20,25) | 59.8 | < 0.001 | 0.144 | < 0.001 | < 0.001 |
| [25,30) | 55 | < 0.001 | 0.02 | < 0.001 | < 0.001 |
| [30,35) | 50.2 | < 0.001 | 0.012 | < 0.001 | < 0.001 |
| [35,40) | 45.4 | < 0.001 | 0.002 | < 0.001 | < 0.001 |
| [40,45) | 40.7 | < 0.001 | < 0.001 | < 0.001 | < 0.001 |
| [45,50) | 36 | < 0.001 | < 0.001 | < 0.001 | < 0.001 |
| [50,55) | 31.5 | < 0.001 | < 0.001 | < 0.001 | < 0.001 |
| [55,60) | 27.1 | < 0.001 | < 0.001 | < 0.001 | < 0.001 |
| [60,65) | 23 | < 0.001 | < 0.001 | < 0.001 | < 0.001 |
| [65,70) | 19.1 | < 0.001 | < 0.001 | < 0.001 | < 0.001 |
| [70,75) | 15.5 | < 0.001 | < 0.001 | < 0.001 | < 0.001 |
| [75,80) | 12.1 | < 0.001 | < 0.001 | < 0.001 | < 0.001 |
| [80,85) | 8.9 | < 0.001 | < 0.001 | < 0.001 | < 0.001 |
| [85,120] | 6.1 | NA | NA | NA | NA |

**Supplemental Table 10: LE comparisons between PWH and general population.**

PWH: People With Hemophilia; LE: Life Expectancies.
